# A cross-sectional analysis of four common clinical decision rules for pulmonary embolism, Mashhad, Iran

**DOI:** 10.34172/jcvtr.32999

**Published:** 2024-09-20

**Authors:** Solmaz Hassani, Neshat Najaf Najafi, Amirhossein Khodadadi, Fahimeh Gandomi, Mahnaz Amini

**Affiliations:** ^1^Endocrine Research Center, Imam Reza Hospital, Mashhad University of Medical Sciences, Mashhad, Iran; ^2^Student Research Committee, School of Medicine, Mashhad University of Medical Sciences, Mashhad, Iran; ^3^Department of Internal Medicine, Imam Reza Hospital, Mashhad University of Medical Sciences, Mashhad, Iran; ^4^Lung Diseases Research Center, Imam Reza Hospital, Mashhad University of Medical Sciences, Mashhad, Iran

**Keywords:** Pulmonary embolism, Diagnostic decision rule, CT angiography

## Abstract

**Introduction:**

Pulmonary embolism (PE) is a potentially fatal condition. Several non-invasive clinical decision rules (CDRs) were developed for the safe exclusion of PE. All CDRs used to safely rule out PE have been created and tested within hospital or acute care environments. However, CDRs that are designed in one specific setting may not perform as effectively when used in a different setting. In this study, we aimed to compare the performance of four common CDRs; Wells Score, Simplified Wells Score, revised Geneva Score, and simplified revised Geneva Score.

**Methods:**

This was a cross-sectional study in which patients suspected of PE presenting to Imam Reza Hospital or Ghaem Hospital were recruited from September 23, 2013, to March 19, 2016 in Mashhad, Iran. The specificity, sensitivity, and accuracy were utilized as metrics to compare the CDRs in our region.

**Results:**

Two hundred and forty patients were included in the study. The mean age of patients was 57.91±19.97 years, and 54.16% of them (n=130) were female. 120 patients were confirmed to have PE with CT angiography. Wells score showed the highest sensitivity (90.4%) and revised Geneva score represented the highest specificity (84.9%). The highest accuracy belongs to the simplified Wells score (62.3%).

**Conclusion:**

In this study, we demonstrated that the Wells criteria with its high sensitivity, can be used as a score for screening, and the revised Geneva score with its high specificity, can be used in the second stage for healthy people who have been diagnosed as unhealthy by the Wells score.

## Introduction

 Pulmonary embolism (PE), is a common clinical problem and can result in significant mortality and morbidity. Acute PE has a notable mortality rate of 30% in untreated cases, but it declines to 8% in diagnosed and treated PE.^1^ Diagnosing PE can be challenging due to the non-specific nature of symptoms and the broad spectrum of clinical presentations among patients with suspected PE, ranging from no symptoms to cardiogenic shock.^2^ Many diagnostic procedures are performed despite the absence of PE. These procedures are costly, time-consuming, and sometimes associated with complications, for instance, the risk of contrast-enhanced computed tomography (CT), including contrast-induced nephropathy, contrast-related allergic reaction, contrast extravasation, and the potential carcinogenic effects of ionizing radiation. Therefore, identifying patients with a low probability of PE allows for appropriate triaging and helps avoid unnecessary referrals or further diagnostic procedures.^3,4^ This risk stratification can be based on either an implicit physician’s estimate known as gestalt or a formal clinical decision rule (CDR). CDRs can improve the effectiveness of the diagnosis by preventing further diagnostic testing for those patients with a low probability of PE. Reducing the number of unnecessary CT scans by 35% is possible by applying this approach, with only 1-2% of cases potentially being missed among patients identified as having a low probability of PE.^5^ Wells score, simplified Wells score, revised Geneva score, and simplified revised Geneva score are the four validated CDRs that have been evaluated in this study. When implementing CDRs in diverse healthcare settings, it is crucial to assess how well they function in each specific environment. This study seeks to evaluate the precision of existing CDRs in our local area and to compare their sensitivity, specificity, and overall accuracy. The goal is to identify the most effective model for predicting the likelihood of PE in our present circumstances.

## Material and Methods

###  Study design and setting

 This was a cross-sectional study conducted in the emergency and inpatient departments of two referral and teaching hospitals (Ghaem and Imam-Reza hospital) in Mashhad, Iran. The date of study was from September 23, 2013, to March 19, 2016. Cases in this study were prospectively recruited and signed a consent form to enter the study. The inclusion criteria were based on the clinical suspicion for PE in patients presented to emergency departments or hospitalised patients in the mentioned hospitals. In addition, cases aged less than 16 or had contraindications for CT angiography were excluded from further investigation. Clinical suspicion for PE consists of patients presenting with acute chest pain, sudden dyspnea, hemoptysis, or unreasonable tachycardia or tachypnea.

###  Study procedure

 Patients enrolled in the study were thoroughly evaluated using history taking, physical examination, and paraclinical procedures. Four resident doctors including two internists, and two cardiologists were responsible for data collection. The collected data for this study includes demographic information, ECG, and CT angiography. All of the data was collected on the first day of admission and interpreted by two relevant specialists in that field who were blinded to the other interpretations. The extent of agreement in the results of the interpretations between two experts was taken into account. If there were conflicts between them, another expert would be included to interpret the data.

###  Definition of clinical decision rules

 Evidence-based literature supports the determination of the pre-test probability of PE before proceeding with further diagnostic procedures. To meet this protocol, different CDRs have been developed to estimate the pre-test probability of PE in suspected patients. Here, we used 4 common CDRs in literature to compare their characteristics in our region.

 The Wells score (Table 1) which was devised in 2000 is among the most commonly used CDRs. Seven variables which were selected out of 40 variables are present in the Wells score. It classifies patients into three or two groups based on their estimated risk of PE (low, moderate, or high risk vs. likely or unlikely). The risk is determined by summing the scores of each variable.^6^ The Simplified Wells score (Table 1) replaced the weighted score for each variable in the original Wells score with a 1-point score for each parameter. It was developed to decrease possible summing mistakes and it is also easier to memorize.^7^ The Revised Geneva score (Table 2), a fully standardized CDR, was developed in 2006 with the aim of removing the implicit judgement of physicians. Hence, this CDR completely relies only on 8 clinical variables.^8^ The Simplified Revised Geneva score (Table 2), which is also understandable from its name, has a 1-point scoring system to reduce possible miscalculations in acute clinical settings.^9^

**Table 1:** The Wells score and simplified Wells score

**Parameters**	**Wells score**	**Simplified Wells score**
Previous PE or DVT	1.5	1
Heart rate > 100 bpm	1.5	1
Recent surgery or immobilisation	1.5	1
Clinical signs of DVT	3	1
Alternative diagnosis less likely than PE	3	1
Haemoptysis	1	1
Cancer	1	1
	Pre-test Probability: ≤ 4: PE unlikely (low) > 4: PE likely (high)	Pre-testProbability: ≤ 1: PE unlikely (low) > 1: PE likely (high)

Abbreviations: DVT: Deep venous thrombosis, PE: Pulmonary embolism, bpm: beat per minute

**Table 2:** The revised Geneva score and simplified revised Geneva score

**Parameters**	**Revised Geneva score**	**Simplified Revised Geneva score**
Age > 65 years	1	1
Active malignancy (or considered cured < 1 year)	2	1
Recent surgery or fracture of the lower limbs within 1 month	2	1
Previous PE or DVT	3	1
Hemoptysis	2	1
Unilateral lower limb pain	3	1
Tenderness on the lower limb, deep venous palpation, and unilateral edema	4	1
Heart rate 75–94 bpm ≥ 95 bpm	3 5	1 2
	Pre-test Probability: < 6: PE unlikely (low) ≥ 6: PE likely (high)	Pre-testProbability: < 3: PE unlikely (low) ≥ 3: PE likely (high)

Abbreviations: DVT: Deep venous thrombosis, PE: Pulmonary embolism, bpm: beat per minute

###  Statistical analysis

 First, the qualitative and quantitative clinical variables were compared between PE and non-PE patients using chi-square and independent-samples t-test. Then, we compared the sensitivity, specificity and accuracy of each CDR. The result of CT pulmonary angiography (CTPA) was the reference value to compare CDRs. Sensitivity is the ratio of patients confirmed by CTPA to have PE who have had high probability of PE. The term specificity refers to the proportion of patients determined not having PE by CTPA who have had low probability of PE. Accuracy is defined as the proportion of correct diagnosis made by CDRs according to CTPA to all made diagnoses. SPSS (Statistical Package for the Social Sciences) version 22 was used and the statistical significance level was set at 0.05.

## Results

###  Demographics and clinical findings

 Two hundred and forty patients were recruited for this study. The mean age of patients was 57.91 ± 19.97 years (range: 17–96 years). Fifty percent of them (N = 120) had PE confirmed by CTPA. Of the cases with PE, 70 (58.33%) were female. Furthermore, among cases with PE, 37 (30.83%) and 11 (9.16%) had a history of previous PE and previous DVT, respectively. The most common initial presentation in cases with PE was dyspnea (N = 103, 85.83%) followed by chest pain (N = 38, 31.66%). Table 3 highlights the clinical and paraclinical findings in patients with confirmed PE and unconfirmed PE.

**Table 3:** Demographic characteristics of subjects

**Variable**	**PE** **(N=120)**	**Not PE** **(N=120)**	***P*** **-value**
Sex (F/M)	70/50 (58.3% / 41.6%)	60/60 (50% / 50%)	0.138
Age (years) Mean (SD)	56.20 (18.42)	59.62 (20.21)	0.172
Previous DVT (N)	11 (9.17%)	3 (2.5%)	0.030*
Previous PE (N)	37 (30.8%)	15 (12.5%)	0.001*
History of cancer (N)	20 (16.7%)	19 (15.83%)	0.415
Bedridden (N)	38 (31.7%)	31 (25.83%)	0.361
History of major surgery (N)	22 (18.3%)	18 (15%)	0.314
History of fracture (N): Lower fracture Upper fracture	5 (4.16%) 3 (2.5%)	7 (5.83%) 4 (3.3%)	0.988 0.694
Tachycardia (N)	68 (56.7%)	110 (91.7%)	< 0.001*
S1Q3T3 (N)	28 (23.3%)	12 (10%)	0.005*
First Presentation (N): Dyspnea Chest pain Hemoptysis Signs of DVT	105 (87.5%) 39 (32.5%) 11 (9.16%) 40 (33.3%)	98 (81.7%) 21 (17.5%) 7 (5.83%) 15 (12.5%)	0.898 0.019* 0.223 0.007*

*Note: *Significant level is < 0.05, chi-square and independent-samples T-test were used Abbreviations: DVT: Deep venous thrombosis, PE: Pulmonary embolism, bpm: beat per minute

###  Clinical decision rules evaluation

 We found that the Wells score shows the highest sensitivity (90.4%) followed by the simplified Wells score (63.5%). The highest specificity is related to the revised Geneva score (84.9%). Simplified Wells score also represents the second highest specificity (61.1%). With regard to the accuracy, the simplified Wells score carries the highest accuracy (62.3%) in comparison to the remaining CPRs (Table 4).

**Table 4:** Characteristics of CDRs

**Type of CDR**	**Sensitivity**	**Specificity**	**Accuracy**
Wells	90.4%	17.1%	54.5%
Simplified Wells	63.5%	61.1%	62.3%
Revised Geneva	22.7%	84.9%	53.8%
Simplified Revised Geneva	60.5%	58.8%	59.7%

Abbreviations: CDR: Clinical decision rule

## Discussion

 PE is associated with a significant morbidity and mortality rate, which points out the importance of prompt diagnosis.^10^ Several CDRs have been designed in order to triage and identify patients with a high probability of this condition and reduce the rate of unnecessary CTs.^11^ Our prospective study compared four CDRs to assess the probability of PE. Based on the meta-analysis by Geersing et al in 2022, the effectiveness of diagnostic approaches for individuals with suspected PE differs significantly among various healthcare environments. The study revealed that all strategies exhibited a sensitivity exceeding 90% in all settings, ranging from 93.3% to 99.6%. However, the specificity of these strategies declined in healthcare settings where PE prevalence was higher, ranging from 7.9% to 67.4%.^12^ In our study, the Wells score, with a sensitivity of 90.4% compared to the other 3 scores was able to better identify patients; therefore, it can be used as a model for screening in this setting. The revised Geneva, score with a specificity of 84.9%, distinguishes healthy people more effectively and can be used as a second stage for healthy people who have been diagnosed as unhealthy by the Wells score. The simplified Wells score with an accuracy of 62.3% had a higher number of accurately predicted values than other models. In a systematic review by Shen et al the diagnostic performance of the Wells score and the revised Geneva score was evaluated. The Wells score demonstrated sensitivity ranging from 63.8% to 79.3% and specificity ranging from 48.8% to 90.0%. In comparison, the revised Geneva score showed sensitivity ranging from 55.3% to 73.6%. The study’s outcomes indicated that the Wells score outperformed the revised Geneva score in distinguishing PE among individuals with suspicion of the condition, which is consistent with our own findings.^13^ Based on a paper by Esiéné et al the Wells score (56.3%) and the simplified Wells score (62.5%) had the highest sensitivity similar to our work. Moreover, the simplified Wells score had the lowest accuracy (37.43%) which was not replicated in our study. Contrarily, the simplified revised Geneva score showed the highest specificity (71.4%).^14^ Wahsh et al showed in their study that the highest sensitivity (92%), specificity (29%) and accuracy (61%) belongs to the simplified wells score, revised Geneva score and simplified wells score, respectively.^15^ To our knowledge, this is the first study in our setting that directly compared 4 widely used CDRs in the diagnostic management of PE prospectively. Nevertheless, this study is subject to certain limitations. Firstly, the sample size and follow-up period were limited. Secondly, the utilization of CDRs along with normal D-dimer levels could potentially provide a safe approach to exclude PE, as indicated in previous research.^16^ However, in our study, we did not assess D-dimer levels. Despite these limitations, given their strong diagnostic performance, both the Wells score and the revised Geneva score remain straightforward and practical methods for stratifying the risk of PE.

 Further studies need to be carried out in order to validate the performance of these CDRs among special populations for instance children, pregnant women, elderly, and also among outpatients in different settings with different PE prevalence like hospitals, clinics, ICUs, or emergency departments. Also, developing artificial intelligence algorithms for future studies may be able to improve patient outcomes through earlier identification of at‐risk patients.^17^

## Conclusion

 In this study, common CDRs capable of PE risk stratification were compared. Our study revealed that the Wells score, with its high sensitivity, can be used as a model for screening, while the revised Geneva score, with its high specificity, can be used in the second stage for healthy people who have been diagnosed as unhealthy by the Wells score.

## Acknowledgments

 We acknowledge the patients for giving their consent in taking part in the study.

## Competing Interests

 Authors declare that there is not any conflict of interests about this study.

## Ethical Approval

 The study was approved by the Institutional Committee of Ethics at Mashhad University of Medical Science (IR.MUMS.MEDICAL.REC.1398.069). Written and informed consent was obtained from all of the participants.
